# The risk factors of hemorrhage in stereotactic needle biopsy for brain lesions in a large cohort: 10 years of experience in a single center

**DOI:** 10.1186/s41016-022-00307-y

**Published:** 2022-12-09

**Authors:** Hailong Li, Chunling Zheng, Wei Rao, Junzhao Sun, Xin Yu, Jianning Zhang

**Affiliations:** 1 Neurosurgery Medical Department, PLA General Hospital, No. 28, Fuxing Road, Haidian District, 100853 Beijing China; 2 Cardiovascular Medical Department, PLA General Hospital, No. 6, Fucheng Road, Haidian District, 100048 Beijing China

**Keywords:** Brain lesion, Complication, Diagnosis, Hemorrhage, Surgery

## Abstract

**Background:**

This study aimed to identify the risk factors for hemorrhage from a large cohort who underwent stereotactic needle biopsy for brain lesions at a single center over a 10-year period.

**Methods:**

We performed a retrospective chart review of consecutive patients who underwent stereotactic biopsy at our institute between January 2010 and December 2019. Demographic characteristics and clinical variables were collected and analyzed to identify risk factors for postbiopsy hemorrhage using the chi-square test and univariable and multivariable logistic regression analyses.

**Results:**

A total of 3196 patients were included in this study; of these, a histological diagnosis was eventually made for 2938 (91.93%) patients. Hemorrhage occurred in 149 (4.66%) patients, and symptomatic hemorrhage occurred in 46 (1.44%) patients. In multivariable logistic regression analyses, the presence of deep-seated lesions (OR 1.272, *p* = 0.035), concomitant edema and enhancement on MR imaging scans (OR 1.827, *p* = 0.002), intraoperative hypertension without a past history (OR 1.012, *p* = 0.024), and the presence of high-grade glioma (OR 0.306, *p* = 0.003) were identified as independent predictors of hemorrhage after biopsy.

**Conclusion:**

Stereotactic needle biopsy is a safe and effective way to obtain tissue from brain lesions for histological diagnosis. The presence of deep-seated lesions, concomitant edema, and enhancement on MR imaging scans and the presence of high-grade glioma are independent predictors of hemorrhage after stereotactic biopsy.

## Background

Stereotactic needle biopsy is a well-established technique for characterizing brain lesions with an uncertain clinical diagnosis or that are unsuitable for microsurgical resection. The procedure can be performed with a frame-based and frameless stereotactic approach, including navigation-assisted and robot-assisted surgery. Stereotactic needle biopsy has been demonstrated to be a relatively safe and highly accurate approach for investigating deep-seated brain lesions and lesions located in special areas, including the sellar region, pineal region, and brainstem. The mortality rate from stereotactic needle biopsy varies from 0 to 4% [1–4], and overall morbidity ranges from 0 to 13% [1, 2, 5–8]. The most common complication associated with stereotactic needle biopsy is intracranial hemorrhage, which can be classified as symptomatic or asymptomatic depending on whether it causes a neurological deficit. Notably, reported perioperative rates of hemorrhage have ranged from 1.3 to 59.8% [1, 5, 6, 9–14]. However, the reasons for this substantial difference in the hemorrhage rate remain uncertain.

Here, we report our experiences from 10 years of stereotactic biopsy at a single center performed with frame-based and frameless surgery and by different operators with different levels of clinical experience. We also investigated the clinical presentations, outcomes—including diagnostic yield—and complications associated with the procedure. In particular, we analyzed the hemorrhage rate and identified risk factors for hemorrhage using univariable and multivariable logistic regression analyses.

## Methods

### Ethics statement

This study design was approved by the institutional ethics committee of our hospital. This work was carried out in accordance with the Declaration of Helsinki. The requirement for informed consent was waived due to the retrospective nature of the study.

### Patient population and definitions

Consecutive patients who underwent stereotactic biopsy in our institute between January 2010 and December 2019 were enrolled in this study. All patients presented with brain lesions of unknown etiology and underwent stereotactic biopsy so that samples of the lesions could be obtained for histological diagnosis. Patients in whom stereotactic procedures were performed for other purposes, such as electrode implantation, drainage, or brachytherapy, were excluded. The lesions of the included patients who were considered unsuitable for direct removal were multifocal, diffuse, or deep-seated, defined as lesions in deep intrinsic cerebral regions that did not involve cortical gray matter, including lesions in the brainstem and other deep-seated locations, such as the basal ganglia region, suprasellar region, and pineal region. All patients underwent routine CT scans within 30 min after surgery. CT scans were performed in the CT room before 2012 and in the operating room after 2012 because mobile CT was available near the operating room. Hemorrhagic complications were defined as the presence of a brain parenchymal hematoma at or around the biopsy site on postoperative CT scans, with or without the development of a new neurological deficit. A high-density area less than 5 mm in diameter observed at the biopsy puncture site on postoperative CT scans was considered an uncertain hemorrhagic complication. The operators were classified as qualified (independently operating in fewer than 200 cases) or experienced (independently operating in more than 200 cases) neurosurgeons. Symptomatic hemorrhage was defined as hemorrhage with newly occurring symptoms directly resulting from intraoperative hemorrhage. A retrospective chart review of the clinical characteristics of the patients, including the sex, age, the use of antiplatelet agents and anticoagulants, prothrombin time, histological diagnosis, method of surgery, operator experience, the occurrence of hemorrhage and complications after stereotactic biopsy, was performed.

### Stereotactic biopsy procedure

Stereotactic biopsy was performed at our center with the following frame-based or frameless navigation-guided systems: a Leksell model G stereotactic frame system (AB Elektra Instruments, Stockholm, Sweden), a manual navigation system (iPlanCranial 2.0 software, BrainLab system, Felkirchen/Munich, Germany), and a robotic system (ROSA robot [ROSA**®**BRAIN, France] and CAS-R-2 robot [Hoz Medical, China]). All patients underwent MR imaging with a slice thickness of 2 mm and CT with a slice thickness of 2.5 mm. The preferred target of biopsy was a substantially enhanced lesion site on T1-weighted MR images or a hyperintense site on T2-weighted MR or fluid-attenuated inversion recovery (FLAIR) images. Surgery was performed under local or general anesthesia. The biopsy trajectories were designed to avoid the sulci and vessels. The dura was punctured with a sharp needle, and tissue samples were then aspirated from different directions using a side-cutting needle with vacuum suction. Compression hemostasis with gel foam soaped with hemopexin that was delivered through an external cannula or external drainage was applied when intraoperative bleeding occurred. After surgery, all patients immediately underwent a CT scan to confirm that the sample site coincided with the target site and to rule out intracranial bleeding.

### Statistical analysis

SPSS 19.0 (SPSS Inc., Chicago, IL) was used for statistical analyses. Categorical variables are expressed as percentages (numbers). Continuous variables are presented as the means ± standard deviations. Statistical tests included nonparametric tests (the Kruskal–Wallis test for continuous variables and the chi-square test and Fisher’s exact test for categorical variables) for evaluating differences among patients with and without hemorrhage who underwent stereotactic biopsy. Univariable and multivariable logistic regression analyses were used to analyze the risk factors that influence hemorrhage. Odds ratios and 95% confidence intervals (CIs) were employed to measure the strength of the associations. A *p* value less than 0.05 was considered to indicate a significant difference.

## Results

**Fig. 1:**
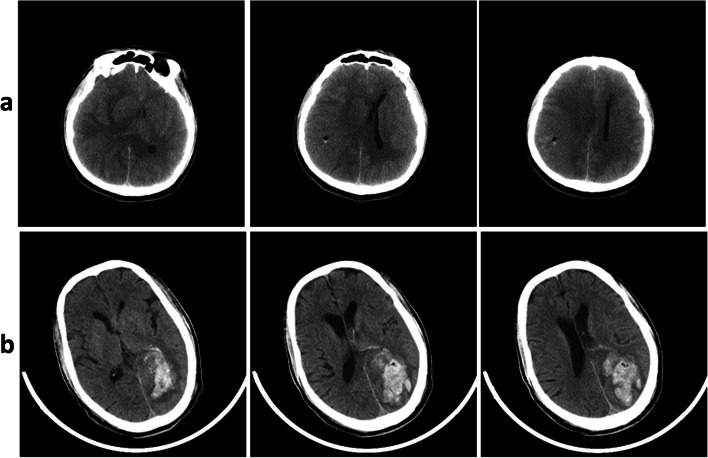
**a** High-density area measuring less than 5 mm in diameter at the biopsy puncture site on the postoperative CT scan was not considered to indicate hemorrhage. **b** Intraparenchymal hematoma measuring more than 5 mm in diameter in the postbiopsy CT scan image was positively defined as operation-related hemorrhage

As shown in Table 1, 3196 patients underwent stereotactic biopsy for histological diagnosis at our center from January 2010 to December 2019. Whether hemorrhage had occurred was uncertain for 45 (1.41%) patients, as these individuals presented with a high-density area measuring less than 5 mm in diameter at the biopsy puncture site on postoperative CT scans (Fig. 1a). A total of 149 (4.66%) patients developed stereotactic biopsy-related hemorrhage (Fig. 1b). Of these patients, 15 (10.07%) underwent craniotomy, and 9 (6.04%) died with or without undergoing craniotomy. A histological diagnosis was made for 2938 (91.93%) patients (Table 2).

**Table 1:** Incidence of hemorrhage and outcomes after stereotactic needle biopsy. Detectable hemorrhage was defined as a hematoma diameter greater than 5 mm regardless of location. Uncertain was defined as a high-density area on CT scan measuring less than 5 mm in diameter

Incidence of hemorrhage	No. of patients(%)	
	Detectable hemorrhage (149/3196, 4.66%)	Uncertain (45/3196, 1.41%)
Location (%)		
Punctuate site only	0 (0)	45 (1.41)
Trajectory restricted	131 (4.10)	0 (0)
Extension to ventricular or subarachnoid space	18 (0.56)	0 (0)
Intraoperative bleeding and treatment (%)	36 (1.13)	33 (1.01)
Symptomatic deficit (%)	46 (1.44)	0 (0)
Craniotomy (%)	15 (0.48)	0 (0)
Death (%)	9 (0.28)	0 (0)

**Table 2:** Demographic characteristics of the patients

Variables	Hemorrhage (*N* = 149, 4.66%)	Nonhemorrhage (*N* = 3047, 95.34%)	*p* value
Diagnostic yield (%)	149 (100)	2789 (71.84)	
Age (means ± standard)	50.72 ± 18.92	47.77 ± 18.22	0.055
Sex (%)			0.790
Male	93 (62.42)	1936 (63.54)	
Female	56 (37.58)	1111 (36.46)	
Location (%)			< 0.001*
Superficial	33 (22.15)	1825 (59.89)	
Deep-seated	116 (77.85)	1222 (40.11)	0.092
Brainstem	21 (18.10)	313 (25.61)	
Other	95 (81.90)	909 (74.39)	
Concomitant edema and enhancement on MR imaging (%)			0.009*
Yes	85 (57.05)	1496 (49.10)	
No	64 (42.95)	1700 (50.90)	
Enhancement on MR imaging alone (%)			0.220
Yes	115 (77.18)	2047 (67.18)	
No	44 (22.82)	990 (32.82)	
Hypertension (%)			0.538
Yes	6 (4.03)	158 (5.19)	
No	143 (95.97)	2899 (94.81)	
Intraoperative hypertension without a past history			0.036
Yes	14 (9.40)	153 (5.02)	
No	135 (90.60)	2894 (94.98)	
Antiplatelet treatment (%)			0.265
Yes	8 (5.37)	111 (3.64)	
No	141 (94.63)	2936 (96.36)	
Prolonged PT (%)			0.138
Yes	10 (6.71)	127 (4.13)	
No	139 (93.29)	2947 (95.87)	
Anesthesia (%)			0.871
Local	140 (93.96)	2835 (93.04)	
General	9 (6.04)	212 (7.96)	
Surgical method (%)			0.608
Frame	102 (68.45)	2135 (70.06)	
Robot-assisted	28 (18.80)	484 (15.88)	
Navigation-assisted	19 (12.75)	428 (14.06)	
Operator experience (%)			0.404
Qualified neurosurgeons	67 (44.97)	1412 (46.34)	
Experienced neurosurgeons	82 (55.03)	1635 (53.66)	
Histological grade of the lesion (%)			0.030*
Benign	14 (9.40)	451 (14.80)	
Malignant	130 (87.25)	2381 (78.14)	
Uncertain	5 (3.35)	215 (7.06)	
Pathology (%)			< 0.001*
High-grade glioma	95 (63.76)	1052 (34.53)	
Lymphoma	28 (16.78)	588 (19.30)	
Metastasis	2 (1.34)	99 (3.25)	
Nondiagnostic	5 (3.36)	253 (8.30)	
Others	19 (14.76)	1055 (34.62)	

*PT*, prothrombin time. Values are number (%) except where indicated otherwise. Special locations include the brainstem, sellar region and pineal region. Qualified neurosurgeons are those who successfully finished fewer than 200 stereotactic biopsies. Experienced neurosurgeons are those who successfully finished more than 200 stereotactic biopsies

^*^Statistically significant difference

### Comparison of the baseline characteristics between patients with and without hemorrhage

The clinical characteristics of all patients were comparatively analyzed and are summarized in Table 2. There was a marginal difference in the mean age between the hemorrhage group and the nonhemorrhage group (50.72 ± 18.92 vs. 47.77 ± 18.22 years, *p* = 0.055). There were no significant differences between the two groups in the following variables: sex (93 [62.4%] vs. 1936 [63.54%], *p* = 0.790), enhancement on MR imaging scans (115 [77.18]) vs. 2047 [67.18%], *p* = 0.220), hypertension with a past history (6 [4.03%] vs. 158 [5.19%], *p* = 0.538), antiplatelet treatment (8 [5.37%] vs. 111 [3.64%], *p* = 0.265), prolonged prothrombin time (PT) (10 [6.71%] vs. 127 [4.13%], *p* = 0.138), anesthesia (140 [93.96%] vs. 2835 [93.04%], *p* = 0.871), surgical method (frame: 102 [68.45%] vs. 2135 [70.06%], *p* = 0.608), and operator experience (67 [44.97%] vs. 1412 [46.34%], *p* = 0.404). Deep-seated lesions were observed in a significantly higher proportion of patients in the hemorrhage group than in the nonhemorrhage group (116 [77.85%] vs. 33 [22.15%], *p* < 0.001), as was concomitant edema and enhancement on MR imaging scans (85 [57.05%] vs. 64 [42.95%], *p* = 0.009) and intraoperative hypertension without a past history (14 [9.40%] vs. 135 [90.60%], *p* = 0.036). Malignant lesions (130 [87.25%] vs. 2381 [78.14%], *p* = 0.030), especially high-grade gliomas (95 [63.76%] vs. 1052 [34.53%], *p* < 0.001), were significantly more common in the hemorrhage group than in the nonhemorrhage group.

### Univariable logistic regression analysis for risk factors that influence hemorrhage

As shown in Table 3, the univariable analysis demonstrated that the variables associated with hemorrhage were age (OR 1.016, *p* = 0.001), the presence of deep-seated lesions (OR 5.243, *p* < 0.001), concomitant edema and enhancement on MR imaging scans (OR 2.131, *p* < 0.001), enhancement on MR imaging scans alone (OR 1.530, *p* = 0.001), intraoperative hypertension without a past history (OR 0.123, *p* = 0.037), and the presence of high-grade glioma (OR 4.368, *p* = 0.041).

**Table 3:** Univariate analysis for risk factors that influence hemorrhage induced by stereotactic biopsy

Factor	Wals	OR	*p* value
Sex	0.077	1.049	0.781
Age	10.243	1.016	0.001*
Deep seated lesions	0.201	5.243	< 0.001*
Concomitant edema and enhancement on MR imaging	18.701	2.131	< 0.001*
Enhancement on radiology	10.918	1.530	0.001*
Hypertension	0.389	0.767	0.531
Intraoperative hypertension without a past history	4.343	0.123	0.037
Antiplatelet treatment	1.113	1.487	0.291
Slightly prolonged PT^#^	0.001	0.101	0.996
Anesthesia	0.185	0.860	0.667
Surgical method	3.686	0.481	0.158
Operator experience	0.113	1.058	0.737
High-grade glioma	4.165	4.368	0.041*

*PT*, prothrombin time

^**#**^Slightly prolonged PT means a PT prolonged by no more than 3 s

^*^Statistically significant

Hemorrhage was not associated with sex (*p* = 0.781), hypertension with a past history (OR 0.767, *p* = 0.531), antiplatelet treatment (OR 1.487, *p* = 0.291), slightly prolonged PT (OR 0.101, *p* = 0.996), anesthesia (OR 0.860, *p* = 0.667), surgical method (OR 0.481, *p* = 0.158), or operator experience (OR 1.058, *p* = 0.737).

### Multivariable logistic regression analysis for independent risk factors predictive of postbiopsy hemorrhage

Stepwise forward multivariable analysis showed that the presence of deep-seated lesions (OR 1.272 95% CI 1.017–1.591, *p* = 0.035), concomitant edema and enhancement on MR imaging scans (OR 1.827, 95% CI 1.242–2.689, *p* = 0.002), intraoperative hypertension without a past history (OR 1.012, 95% CI 1.002–1.457, *p* = 0.024), and the presence of high-grade gliomas (OR 0.306, 95% CI 0.139–0.879, *p* = 0.003) were independent risk factors associated with hemorrhage after stereotactic biopsy. Sex (*p* = 0.797), age (*p* = 1.030), antiplatelet treatment (*p* = 0.839), slightly prolonged PT (*p* = 0.322), anesthesia (*p* = 0.796), surgical method (*p* = 0.199), operator experience (*p* = 0.347), and hypertension (*p* = 0.118) remained unassociated with hemorrhage after controlling for other variables (Table 4).

**Table 4:** Multivariate logistic regression analysis for risk factors that influence hemorrhage

Factor	OR	95% CI	*p* value
Deep-seated lesion	1.272	1.017–1.591	0.035*
Concomitant edema and enhancement on MR imaging	1.827	1.242–2.689	0.002*
Intraoperative hypertension without a past history	1.012	1.002–1.457	0.024*
High-grade glioma	2.306	1.139–4.874	0.003*
Sex	/	/	0.797
Age	/	/	1.030
Antiplatelet treatment	/	/	0.839
Slightly prolonged PT	/	/	0.322
Anesthesia	/	/	0.796
Surgical method	/	/	0.199
Operator experience	/	/	0.347
Hypertension with a past history	/	/	0.118

*PT*, prothrombin time

^**#**^Slightly prolonged PT means that PT prolonging was no more than 3 s

^*^Statistically significant

### Symptomatic hemorrhage was associated with lesions in certain locations

A comparative analysis was performed between the symptomatic hemorrhage group and the asymptomatic hemorrhage group. There was no significant difference in mean age between the two groups (50.19 ± 21.00 vs. 51.21 ± 18.32 years, *p* = 0.802) (Table 5). There were no significant differences between the two groups in the following variables: concomitant edema and enhancement on MR imaging scans (*p* = 0.472), histological grade (*p* = 0.991), and pathology (*p* = 0.773). Deep-seated locations (*p* < 0.001), hemorrhage diameter (*p* < 0.001), and hemorrhage extending pattern (*p* < 0.001) were associated with significant symptomatic hemorrhage.

**Table 5:** Risk factors associated with symptomatic hemorrhage

Factor	Symptomatic hemorrhage (*n* = 46)	Asymptomatic hemorrhage (*n* = 103)	*p* value
Age (%)	50.19 ± 21.00	51.21 ± 18.32	0.802
Location of lesions (%)			
Superficial location	17 (36.96)	87(84.47)	< 0.001*
Deep-seated location	29 (63.04)	16(15.53)	
Brainstem	20 (68.96)	5(31.25)	0.027
Other	9 (31.04)	11 (68.75)	
Hemorrhage diameter			
5 ~ 50 mm	5 (10.87)	95 (92.23)	< 0.001*
> 50 mm	41 (89.13)	9 (7.77)	
Hemorrhage extending pattern			
Restricted in trajectory	30 (65.22)	101 (98.06)	< 0.001*
Extension to ventricular or subarachnoid space	16 (34.78)	2 (1.94)	
Concomitant edema and enhancement on MR imaging (%)			0.472
Yes	30 (65.22)	60 (58.25)	
No	16 (34.78)	43 (41.75)	
Histological grade (%)			0.991
Benign	5 (10.87)	11 (10.68)	
Malignant	39 (84.78)	87 (84.47)	
Uncertain	2 (4.35)	5 (4.85)	
Pathology (%)			0.773
High-grade glioma	34 (73.90)	65 (63.11)	
Lymphoma	4 (8.70)	13 (12.62)	
Metastasis	4 (8.70)	11 (10.68)	
Nondiagnostic	2 (4.35)	6 (5.83)	
Others	2 (4.35)	8 (7.76)	

^*^Statistically significant

### Death was more likely in patients with malignant tumors

As shown in Table 6, there were 9 deaths (6.04%) among patients with or without craniotomy decompression. Pathologically, high-grade gliomas were observed in the majority of these patients. Refractory brain edema, central nervous system infection, and rebleeding were the main causes of death.

**Table 6:** Deaths among the study cohort

No	Pathology	Hematoma volume	Hemorrhagic extension	Treatment	Cause of death
1	Lymphoma	28 ml	/	Craniotomy	CNS infection
2	High-grade glioma	43 ml	/	Craniectomy	Refractory brain edema
3	High-grade glioma	35 ml	/	Craniectomy	Refractory brain edema
4	High-grade glioma	48 ml	/	Craniectomy	CNS infection
5	Lymphoma	39 ml	/	Craniectomy	Bebleeding
6	Germinoma	/	Into arachnoid space	EVD and conservation therapy	Refractory brain edema
7	High-grade glioma	45 ml	/	Craniectomy	CNS infection
8	High-grade glioma	55 ml	/	Craniectomy	Rebleeding
9	Choriocarcinoma	/	Into arachnoid space	EVD and conservation therapy	Refractory brain edema

*EVD*, external ventricular drainage; *CNS*, central nervous system

## Discussion

Hemorrhage is the most common perioperative complication after a stereotactic needle biopsy. Regardless of whether the stereotactic biopsy is performed with a frame-based or frameless method, the joint incidence of symptomatic and asymptomatic hemorrhage ranges from 1.3 to 59.8%. The hemorrhage rates for stereotactic needle biopsies reported in major published studies are listed in Table 7. To identify the independent risk factors that potentially influence the hemorrhage rate, several relevant questions were investigated and the statistical results were assessed.

**Table 7:** Review of the literature on hemorrhagic complications and analysis of risk factors for hematoma after stereotactic biopsy for brain lesions

Author and year	No. of cases	Hemorrhage incidence	Independent risk factors
Kulkarni et al. (1998) [9]	102	59.8%	Malignant glioma
Field et al. (2001) [2]	500	8%	Platelet count less than 150,000 mm
Grossman et al., (2005) [15]	355	7%	Lesion located in the brainstem
McGirt et al. (2005) [7]	270	9%	Diabetes mellitus, thalamic lesion, basal ganglion lesion
Shakal et al. (2014) [6]	147	4.7%	Malignant tumor
Malone et al. (2015) [5]	7514	5.8%	Older age; hydrocephalus; edema
Beynon et al. (2018) [14]	159	1.3%	/
Mizobuchi et al. (2019) [1]	80	31.3%	Prothrombin time longer than 12.7 s; malignant glioma
Taweesomboonyat et al. (2019) [12]	85	19%	Lesion diameter 3 cm or smaller
Present study	3196	4.66%	Deep-seated lesion; concomitant edema and enhancement on radiology; malignant glioma

In each series, the characteristics for identifying new hemorrhages on postoperative CT scans and the definitions of the symptoms of new neurological deficits were variable, not clearly stated, or inconsistently counted. We reviewed the associated references and concluded that these substantial differences may contribute to the wide range of reported hemorrhage rates. Kulkarni et al. [9] classified new hemorrhages by location and size and found that 23 (41.1%) of the 56 hemorrhages that were intraparenchymal measured less than 5 mm in maximum diameter. Mizobuchi et al. [1] reported that hemorrhages were smaller than 5 mm in diameter in 22 (88%) of 25 patients with intraparenchymal biopsy-related hemorrhages. Shakal et al. [6] defined only the location, and not the diameter, of new hemorrhages. In our study, a high-density area measuring less than 5 mm in diameter at the puncture site of biopsy on postoperative CT scans was not considered a hemorrhagic complication. Consistent with McGirt’s [7] opinion, we determined such findings to be merely a normal change after stereotactic surgery, not a complication.

Another factor contributing to the differences in the reported hemorrhage incidence rate may be the management of intraoperative bleeding after the stereotactic biopsy. Shakal et al. [6] suggested allowing blood to flow from the cannula unimpeded when intraoperative bleeding occurs. Mizobuchi et al. [1] recommended the insertion of a silicon drainage tube with an outer diameter of 2.5 mm to prevent postoperative hemorrhage. De Quintana-Schmidt et al. [10] applied a thrombin-gelatin matrix for the management of intractable hemorrhage during the stereotactic biopsy. In their study, immediate hemostasis was achieved in all 3 (100%) patients receiving a matrix injection. A postoperative CT scan showed that 1 (33%) patient presented with a high-density area less than 5 mm in diameter at the biopsy puncture site. Based on our experience, we recommend compression hemostasis with gelfoam soaped with hemopexin delivered through the external cannula when intraoperative bleeding occurs. Interestingly, 45 patients (shown in Table 1) had a high-density area measuring less than 5 mm at the biopsy puncture site on postoperative CT. These results demonstrated that the management of intraoperative hemostasis probably influenced the visibility of the hemorrhage on postoperative CT scans.

After identifying the subjective differences between groups, we found that age, the presence of deep-seated lesions, concomitant edema and enhancement on MR imaging scans, enhancement on MR imaging scans alone, intraoperative hypertension without a past history, and the presence of malignant glioma were associated with hemorrhage in univariable logistic regression analysis. The stepwise forward multivariable analysis further revealed that the presence of deep-seated lesions (OR 1.272), concomitant edema and enhancement on MR imaging scans (OR 1.827), intraoperative hypertension without a past history (OR 1.012), and the presence of high-grade glioma (OR 2.306) were independent risk factors associated with hemorrhage after stereotactic biopsy. These findings are in accordance with the results of many previous studies [1, 5–7, 9, 15]. Regarding the correlation between hemorrhage and the location of the lesion, there is little difference between the findings reported in the present study and those described in previous studies. Grossman et al. correlated the risk of hemorrhage with lesions located in the brainstem [15]. McGirt et al. [7] correlated the risk of hemorrhage with lesions located in the thalamus and basal ganglia. Field et al. revealed that the risk of bleeding from hematomas measuring more than 5 mm in diameter increased by a factor of 5.1 following the biopsy of lesions located in the pineal region. Moreover, to explore whether there was a difference in the risk of hemorrhage between lesions located in the brainstem and other deep-seated locations, we analyzed these two groups of lesions separately. Interestingly, the brainstem was not found to carry a higher risk of hemorrhage following stereotactic biopsy in our previous experience [16], in the present study (Table 2, *p* = 0.092), or in other studies [17, 18]. However, hemorrhage in the brainstem led to a higher rate of neurological deficits (Table 5, *p* = 0.027). This difference was attributed to the fact that biopsied lesions in other deep-seated locations are pathologically associated with malignant gliomas, whereas biopsied lesions in regions of the brainstem, such as the pons, are associated with low-grade gliomas, especially in children.

The presence of a malignant tumor and concomitant edema and enhancement on MR imaging scans were independent and correlative predictors of hemorrhage following biopsy. Compared with other lesions, high-grade gliomas were significantly more common in the hemorrhage group than in the nonhemorrhage group and led to death. Similarly, concomitant edema and enhancement on MR imaging scans were observed in a significantly higher proportion of patients in the hemorrhage group than in the nonhemorrhage group (85 [57.05%] vs. 64 [42.95.10%]). Additionally, intratumoral hemorrhage was the most common type of hemorrhage related to a stereotactic needle biopsy, consistent with previous studies [1, 5, 6]. The presence of concomitant edema and enhancement on MR imaging scans has been widely used to assess the perfusion and grade of gliomas and tumor angiogenesis [19–21]. These results demonstrated that the probability of bleeding after biopsy is increased in highly malignant lesions with abnormal vessels. Interestingly, intraoperative hypertension without a past history was significantly associated with hemorrhage regardless of the statistical method used. This information serves as a reminder for surgeons to focus more attention on controlling intraoperative blood pressure. Hence, careful preparation, optimal selection of the target site, and intraoperative management should be ensured during stereotactic needle procedures for high-grade gliomas.

Although the surgical plan and operator experience are very important in stereotactic biopsy procedures, the surgical method and operator were not correlated with the incidence of hemorrhage. In a meta-analysis, frame-based and frameless intracranial stereotactic biopsy procedures were both confirmed to be safe and efficient, and the two techniques did not present significant differences in diagnostic yield, biopsy-related morbidity, or biopsy-related mortality [11]. For the joint frame-based and frameless biopsy cohort, the calculated OR for hemorrhagic complications after the biopsy was 1.16, suggesting no significant differences in the incidence of postbiopsy hemorrhage between the two types of techniques. Our results showed that there was no significant relationship between the surgical method and operator and the risk of hemorrhage during stereotactic needle biopsy procedures. These observations provide evidence indicating that an appropriate surgical plan and a standardized operation procedure can guarantee the success of stereotactic biopsy.

**Fig. 2:**
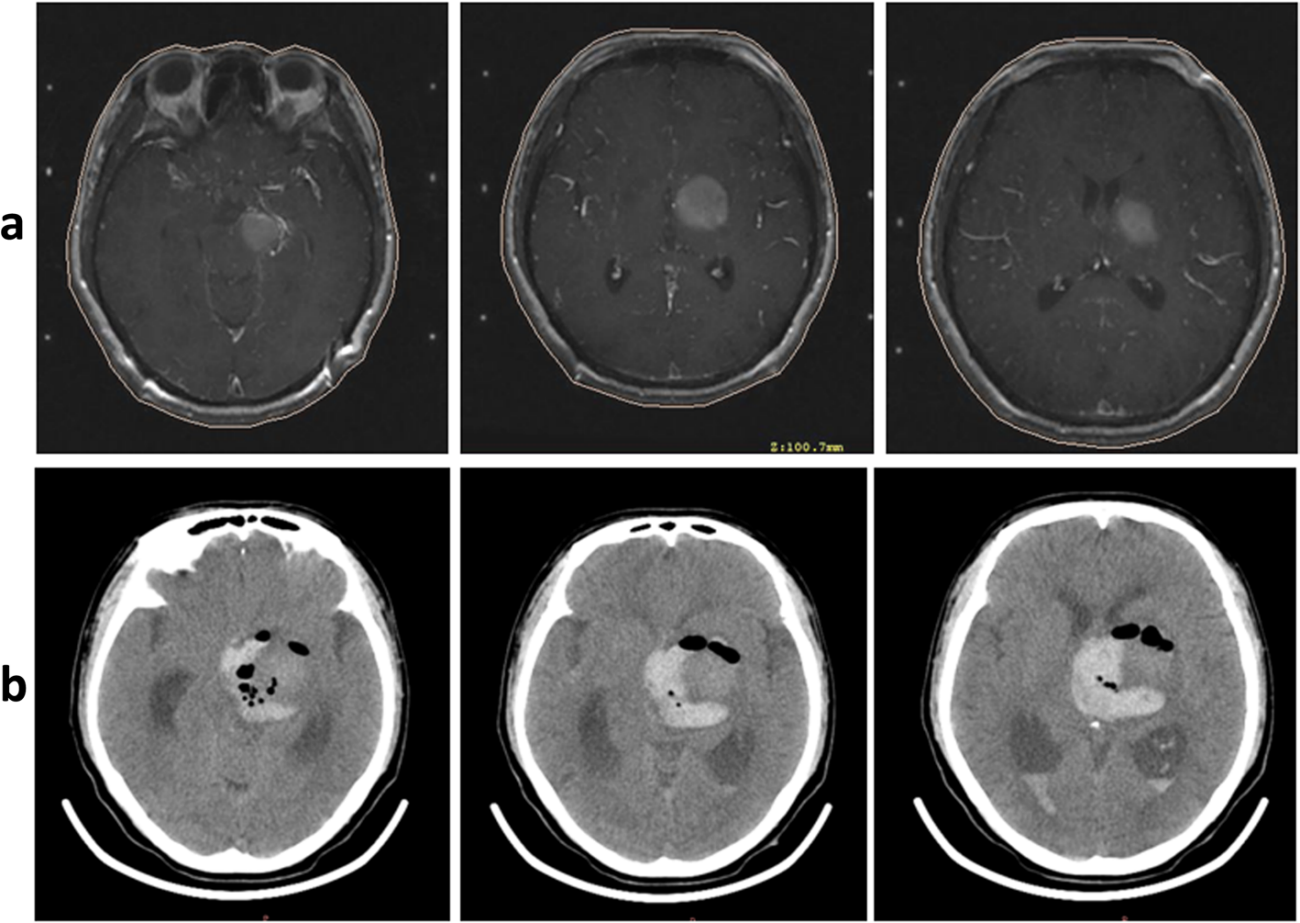
**a** Stereotactic postcontrast MR imaging scan demonstrated a left thalamoganglionic mass. **b** Postoperative CT scan showed intraparenchymal hemorrhage with intraventricular extension resulting from a prothrombin time longer than 15 s

In contrast to some series reporting a statistical relationship between the risk of hemorrhage and coagulation and platelet count [1, 2], hemorrhage was not correlated with coagulation effects in our study. However, we strongly recommend that potential coagulation abnormities be corrected before surgery. One patient with a prothrombin time longer than 15 s in our study developed an intraoperative intraparenchymal hematoma despite a negative bone marrow biopsy; pathology confirmed diffuse large B cell lymphoma, and unfortunately, the patient died (Fig. 2). As a result, we no longer perform a stereotactic biopsy on patients with abnormal coagulation profiles.

## Conclusion

Stereotactic needle biopsy is a feasible, safe, and effective method for obtaining samples of brain lesions for histological diagnosis. The presence of deep-seated lesions, concomitant edema and enhancement on MR imaging scans, intraoperative hypertension without a past history, and the presence of malignant glioma are independent predictors for hemorrhage after stereotactic biopsy. Furthermore, perioperative hemorrhage does not appear to be correlated with the type of navigation used or operator experience. Therefore, future research on this topic should focus on preoperative assessments, related advancements, and the intraoperative management of hemostasis.

## Data Availability

The datasets generated during the current study are not publicly available due to military secrecy but are available from the corresponding author on a reasonable request.

## Abbreviations

CI: Confidence interval
CT: Computed tomography
FLAIR: Fluid-attenuated inversion recovery
MR: Magnetic resonance
OR: Odds ratio
PT: Prothrombin time
